# Differences in the Characteristics of Barrett's Esophagus and Barrett's Adenocarcinoma between the United States and Japan

**DOI:** 10.1155/2013/840690

**Published:** 2013-03-28

**Authors:** Makoto Oryu, Hirohito Mori, Hideki Kobara, Noriko Nishiyama, Shintaro Fujihara, Mitsuyoshi Kobayashi, Mitsugu Yasuda, Tsutomu Masaki

**Affiliations:** Department of Gastroenterology and Neurology, Faculty of Medicine, Kagawa University, 1750-1 Ikenobe, Miki-cho, Kita-gun, Takamatsu, Kagawa 761-0793, Japan

## Abstract

In Europe and the United States, the incidence of esophageal adenocarcinoma has increased 6-fold in the last 25 years and currently accounts for more than 50% of all esophageal cancers. Barrett's esophagus is the source of Barrett's adenocarcinoma and is characterized by the replacement of squamous epithelium with columnar epithelium in the lower esophagus due to chronic gastroesophageal reflux disease (GERD). Even though the prevalence of GERD has recently been increasing in Japan as well as in Europe and the United States, the clinical situation of Barrett's esophagus and Barrett's adenocarcinoma differs from that in Western countries. In this paper, we focus on specific differences in the background factors and pathophysiology of these lesions.

## 1. Introduction

With the increased prevalence of gastroesophageal reflux disease (GERD), the incidence of Barrett's esophageal adenocarcinoma (BEA) derived from Barrett's esophagus (BE) has been increasing dramatically in Europe and the United States since the 1980s and currently accounts for 60–70% of all esophageal cancers, having surpassed squamous cell carcinoma [1]. In the United States, interest in BE, the source of BEA, is high because of the explosive increase in BEA cases, despite a decrease in the overall incidence of cancer. In Japan, >90% of esophageal cancer is squamous cell carcinoma, with adenocarcinoma accounting for <1-2% [2]. However, as in Western countries, the incidence of GERD in Japan has been increasing in recent years because of Westernized eating habits and a decline in *Helicobacter pylori* infection in young people owing to improved hygienic environments. Therefore, even with no noticeable increase in the rate, there is a concern over the imminent increase in BEA in Japan. Nonetheless, it is currently unclear whether BEA will increase in the same fashion as it has in Europe and the United States. In this paper, we comprehensively review reports on BE and BEA and describe the current clinical situation.

## 2. Diagnosis of Barrett's Esophagus

**Figure 1:**
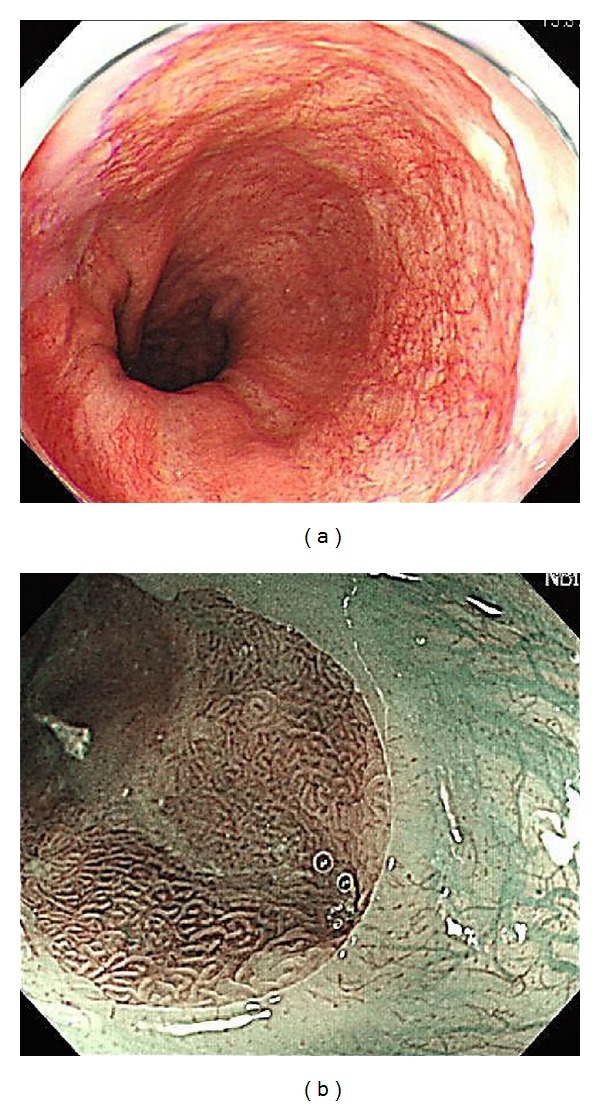
(a) The esophagogastric junction was diagnosed in the upper end of the gastric fold and the lower end of the palisade vessels. Palisade vessels can be found in the Barrett esophagus. (b) Barrett esophagus by magnifying endoscpy and narrow band Imaging (NBI) observation.

**Figure 2:**
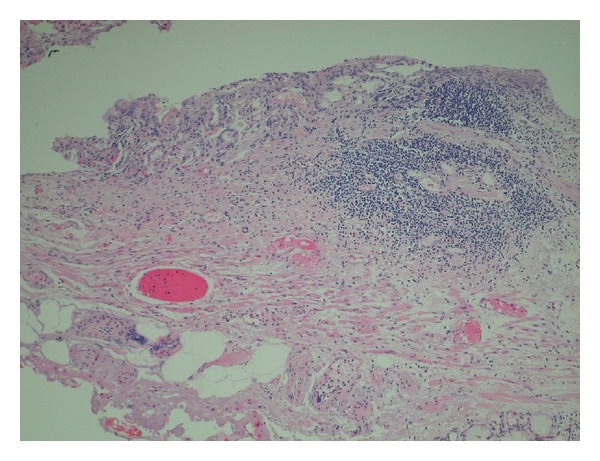
Histopathological findings of the resected specimen (low-power view). Low-power view shows the remained squamous epithelium, specialized columnar epithelium, and esophageal gland or duct.

**Figure 3:**
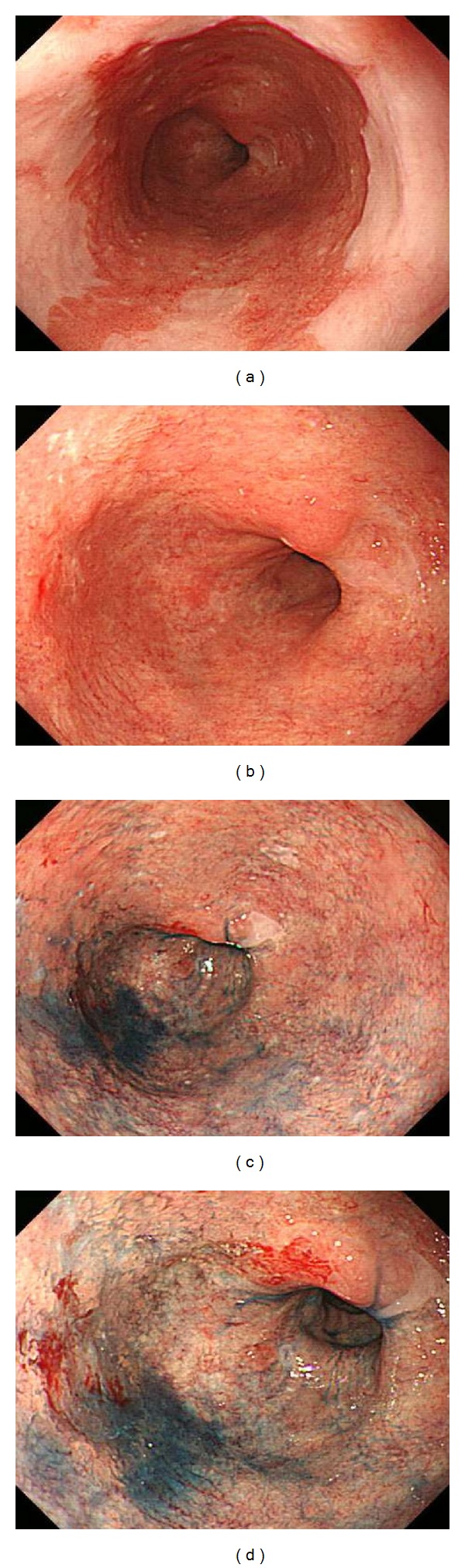
(a) Squamous columnar junction is located at 29 cm from the incisors. (b) Mucosal changes are seen in the entire circumference of pale redness and rough from squamous columnar junction to upper end of the gastric fold. (c) and (d) Endoscopic view revealed a reddish protruded lesion with an uneven surface located at 1 o'clock position in the oral side of Barrett's esophagus after the spreading of indigo carmine.

The diagnosis of BE has undergone changes unique to each country since the first report by Norman Rupert Barrett in 1950. Regardless of lesion length, BE in Europe and the United States is subject to endoscopic examination followed by histological biopsy. BE is defined as a transformation of esophageal epithelium into specialized columnar epithelium including goblet cells, thus making histological examination essential in the diagnosis of BE [3]. This is particularly important because the specialized columnar epithelium is believed to play a powerful role in the malignant conversion of BE (Figures 1, 2, and 3). However, Vieth et al. did not observe goblet cell metaplasia in the background mucosa in 56.6% of their patients with BE and thus recommended the exclusion of goblet cell metaplasia from the diagnostic criteria of BE [4]. On the other hand, the Japanese Society for Esophageal Diseases regards BE as columnar-lined esophagus (CLE) that is simply continuous from the stomach and does not require the histological evidence of intestinal metaplasia for diagnosis. Similarly, the guidelines for BE diagnosis of the British Society of Gastroenterology (BSG) state that “the presence of areas of intestinal metaplasia, although often present, is *not* a requirement for diagnosis” [5]. Similarly, a recent report in North America recommends the exclusion of intestinal metaplasia from the definition of BE [6]. For all of these reasons, we await global standardization of the histological criteria for BE.

Moreover, to standardize diagnostic endoscopy for BE, the International Working Group for the Classification of Oesophagitis recommends classification based on the Prague C & M criteria [7], which make a clear distinction between endoscopic BE and histologic BE by defining the circumferential length of BE as C (circumferential extent) and that extending toward the mouth as M (maximum extent). The proximal end of the gastric longitudinal folds is regarded as the esophagogastric junction and serves as an endoscopic landmark. However, this landmark is affected by respiratory and peristaltic movement, and the longitudinal folds are difficult to observe in the case of severe gastric atrophy caused by *H. pylori* infection, which is prevalent in Japan. Although the distal end of the lower esophageal palisade vessels observed under deep inspiration is generally regarded as the esophagogastric junction in Japan, it is difficult to observe palisade vessels in the presence of severe inflammation or atypical lesions. Moreover, in endoscopic diagnosis using the C & M criteria, the reliability coefficient (RC) for BE > 1 cm was 0.72, but the RC for BE < 1 cm was as low as 0.21 [8].

Therefore, not only the histological diagnostic criteria for BE but also the esophagogastric junction used as an endoscopic landmark remain nonuniformly defined. Furthermore, compared with large segment BE (LSBE), short segment BE (SSBE), particularly ultra-SSBE (<1 cm), has low endoscopic diagnostic accuracy, and this is likely the cause of the variability in the prevalence of BE between different facilities and countries.

## 3. Pathogenesis of Barrett's Esophagus

BE is a disorder in which esophageal squamous epithelial cells are replaced by columnar cells. This is presumably because squamous cells damaged by repeated acid reflux are replaced by columnar epithelium, which is more resistant to stomach acid. In Europe and the United States, BE is present in 5–15% of GERD patients, and the rate reportedly increases in patients with higher frequency and longer duration of acid reflux [9]. A previous study conducted in Japan reported that the location of BE and BEA coincides with the site of GERD [10]. These two studies strongly suggest an association between BE and stomach acid reflux. Moreover, a positive correlation has been reported between the length of BE lesion and the duration of exposure to acid in the esophagus [11], indicating that stomach acid refluxed into the esophagus is involved in both the development and length of BE.

On the other hand, a previous study found no correlation between the prevalence of BE and symptoms of heartburn or the presence of GERD [12], while another study reported the development of BE in individuals who had undergone total gastrectomy and thus lacked stomach acid [13]. These studies cast doubt on the role of repeated stomach acid reflux in the gradual enlargement of BE and suggest the involvement of other causal factors.

A mixture of stomach fluid and duodenal fluid is more toxic to the esophageal mucosa than either fluid alone [14], suggesting that the cytotoxicity of stomach acid is enhanced by bile acid. In addition, compared with GERD patients without complications, the frequency of bile acid reflux and the concentration of bile acid in the esophagus are high in BE patients [15, 16]. Moreover, the expression of transcription factors—the caudal-type homeobox genes CDX1 and CDX2—in the intestine is influenced by bile acid more than by stomach acid [17, 18]. It is therefore possible that bile acid is also involved in the onset of BE and that bile acid not only damages the esophageal mucosa but also directly induces intestinal metaplasia.

It has been widely accepted that obesity is a risk factor for GERD [19]. In severely obese individuals (body mass index > 35.0), increased abdominal pressure is thought to cause the reflux of duodenal fluid into the stomach, leading to the reflux of stomach fluid containing duodenal fluid into the esophagus.

## 4. Enlargement of Barrett's Esophagus

BE is classified as LSBE if the length of BE extends >3 cm from the esophagogastric junction and as SSBE if the length is <3 cm or if it is noncircumferential. SSBE has always been considered the precursor to LSBE, with tongue-shaped SSBE gradually enlarging into LSBE [20]. Cameron and Lomboy, however, did not observe any significant change in the length of BE after a 7-year follow-up observation of 50,000 patients, and age at onset had no effect on length. They therefore concluded that the length of BE is predetermined at onset and does not increase [21].

In Japan, Manabe et al. investigated 500 SSBE cases indicated for endoscopy, and, after a mean observation period of 5.7 years, they reported that 477 (95.4%) patients had BE < 3 cm, 23 (4.6%) had BE ≥ 3 cm, 263 (52.6%) had esophageal hiatal hernia, and 52 (10.4%) had GERD. No complications involving adenocarcinoma were observed during the study period. However, enlargement of SSBE was observed in 29 (5.8%) cases, and, in many of these cases, BE did not grow at a constant rate but extended rapidly during a certain period of time. The length of BE at onset was <1, 1–3, and ≥3 cm in approximately 2%, 10%, and 17% of the 29 cases, respectively [22]. Therefore, even though most cases of SSBE do not show growth during the clinical course, the length at onset appears to influence the growth in some cases of SSBE. Factors promoting the growth of BE involve complications with GERD, esophageal hiatal hernia, mild atrophy of gastric mucosa, length of BE at onset ≥1 cm, and non-circumferential flame-shaped morphology [22, 23]. Compared with SSBE, LSBE has a strong association with cancer [24], and because a small population of SSBE cells can develop into LSBE, prevention of BE enlargement is important in terms of oncogene suppression. Before suppression can be performed, however, it is necessary to clearly identify cases of SSBE with the potential to transform into LSBE.

## 5. Prevalence of Barrett's Esophagus

In Europe and the United States, the prevalence of BE in GERD patients is 10–15% [25], and the rate of LSBE with intestinal metaplasia ≥3 cm is approximately 5% [26–31], while that of SSBE is 6–12% [32–35]. In Japan, an endoscopic study of 1668 subjects revealed that the prevalence of Barrett's mucosa ≥5 mm is 0.2% and 37.7% in endoscopic LSBE and SSBE and 0.2% and 19.9% in histologic LSBE and in SSBE with specialized columnar epithelium, respectively [36]. Although the prevalence of BE in Japan varies between studies, the general consensus is that the prevalence of LSBE and SSBE is <1% and ≥96%, respectively, revealing a significantly lower prevalence of LSBE in Japan compared with Western countries.

The key question then is “what factors contribute to the huge gaps in the prevalence of SSBE and LSBE between the United States and Japan?” This phenomenon cannot be fully explained by differences in endoscopic and histological diagnostic criteria. Simple classification of the background mucosa of BE reveals that many BE cases in Japan are SSBE < 3 cm formed mainly by the mucosa of gastric cardiac glands. On the other hand, BE cases observed in the United States are mostly LSBE ≥ 3 cm formed by esophageal specialized columnar epithelium.

The transcriptional factors CDX1 and CDX2 in the intestine are affected more by bile acid than by stomach acid [17, 18], and, in the United States where the intake of fat is greater than in Japan, severe obesity may increase the frequency of duodenal fluid reflux containing bile acid, thus promoting the onset of LSBE.

When we compared the contribution of GERD to the development of BE between the United States and Japan, the results revealed that the prevalence of reflux esophagitis in Japan is approximately 10% and is increasing, as in the United States [37]. In a study grading cases in Japan using the Los Angeles Classification of GERD severity, 55%, 32%, and 13% of cases were grade A, B, C+D, indicating low severity [38]. Therefore, although the prevalence of GERD is increasing in Japan, as in Western countries, the severity of GERD in Japan remains relatively low, which may contribute to the difference in the prevalence of LSBE.

Among a large number of studies reporting the association between GERD and *H. pylori*, meta-analysis of 20 studies investigating the rate of *H. pylori* infection in GERD patients revealed a rate of 38.2% in GERD patients and 49.5% in controls, with an odds ratio of 0.6 (95% confidence interval, 0.47–0.78). The rate of *H. pylori *infection was therefore significantly lower in GERD patients [39]. The relationship between GERD and *H. pylori* appears to vary by region, and the rate of *H. pylori* infection in GERD patients has been low in all studies conducted in East Asia, with North America showing a similar trend, even though studies conducted in Europe have shown no difference in the infection rate between GERD patients and controls.

In 2000, the rates of *H. pylori* infection in Japanese GERD patients and healthy individuals older than 60 years were 24% and 83%, respectively, thus showing a significantly lower rate in GERD patients (*P* < 0.01). On the other hand, in GERD patients and healthy individuals younger than 59 years, the rates of *H. pylori* infection were 49% and 64%, with no significant difference between age groups. The prevalence of *H. pylori *infection is high among the elderly in Japan, and atrophy of the gastric mucosa due to *H. pylori* infection appears to cause a functional decline in gastric secretion. Because the rate of *H. pylori *infection has been declining rapidly in younger generations, the overall infection rate will definitely decrease in Japan as has been seen in Western countries.

With regard to the earlier posed question as to why the prevalence of SSBE and LSBE varies to such an extent between the United States and Japan, even though GERD is the cause in both SSBE and LSBE, it may be necessary to study the pathophysiology of SSBE and LSBE separately because their pathogenesis may differ due to the different clinical backgrounds, such as the severity of GERD and the composition of refluxed fluid. It is currently unclear whether Japan will simply follow the same path as that in Europe and the United States, which is characterized by an increase in obesity and a decrease in *H. pylori* infection.

## 6. Pathogenesis of Barrett's Adenocarcinoma

BE is an important source of BEA, and there is no doubt that prolonged GERD plays a strong role in the transformation of BE to BEA. Previous studies have shown that refluxed fluid, particularly stomach and bile acid, is deeply involved in the pathogenesis of BE [40, 41]. Inflammation of the esophageal mucosa due to chemical stimuli generated by refluxed stomach and bile acids may be an indirect causal factor of BE. In experiments using cultured squamous epithelial cells, stomach and bile acids induced the expression of CDX2, an inducer of transcription factors specific to the intestinal tract [40], demonstrating the direct functional involvement of these acids in the pathogenesis of BE. Furthermore, a study using an acid-reflux animal model reported that a high-fat diet consisting mainly of beef fat resulted in the modulation of bile acid fractions and subsequently promoted the transformation of BE to BEA [42]. This suggests that, in addition to the proportion of bile acid in refluxed digestive fluid, a change in the composition of bile acid is also involved in the pathogenesis of BEA. Therefore, together with the increased cell proliferation seen in Barrett's mucosa, a wide range of DNA damage caused by prolonged chronic inflammation due to the reflux of stomach and duodenal acid that persists even after the formation of BE appears to increase oncogenic risk [43].

## 7. Risk of Developing Barrett's Adenocarcinoma from Barrett's Esophagus

**Figure 4:**
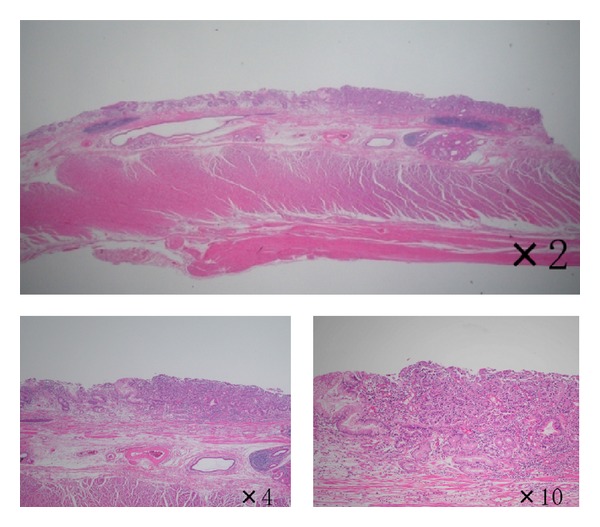
Histopathological findings of the resected specimen (low-power view). Pathological diagnosis shows Barrett's adenocarcinoma close to squamous epithelium.

In Europe and the United States, the incidence of esophageal adenocarcinoma has increased sixfold in the last 25 years, drawing new attention to the disease as one of the highest increasing rate of the cancers [44]. This rapid increase in esophageal adenocarcinoma is believed to be caused by the increased prevalence of BE accompanying GERD and by the reduced rate of *H. pylori* infection [45, 46]. In Japan, the incidence of GERD has been increasing rapidly since the late 1990s, with a similar trend in the incidence of BEA. However, compared with squamous cell carcinoma, which accounts for >90% of all esophageal cancers, the proportion of adenocarcinoma remains around 1-2%, with no significant increase [2] (Figures 3 and 4).

BE is a precancerous lesion that can develop into BEA via a metaplasia → dysplasia → carcinoma sequence [47], which is the basis for the diagnostic criteria defining specialized intestinal metaplasia, including goblet cells, as BE in the United States. Although the risk of BE transforming into BEA is about 0.5% [48–50], one study showed that adenocarcinoma developed within 5 years in approximately 20% and 50% of BE patients with low- and high-grade dysplasia, respectively [51]. Therefore our future challenge will be to improve the prevention of dysplasia and adenocarcinoma at the metaplasia stage.

In a number of studies investigating the risk factors for BE, the length of BE was reported as the common risk factor. According to Weston et al., the risk of conversion into high-grade dysplasia or carcinoma increases 1.39-fold as the length of BE increases by 1 cm [52]. Similarly, in a recent study, cancer incidence increased by 24% as the length of BE increased by 1 cm [53]. Furthermore, in a mean 5.5-year follow-up study of 1204 patients with BE, Wani et al. observed no cancer development in patients with BE < 2 cm and thus reported that SSBE has an extremely low oncogenic risk [54]. However, BEA is frequently associated with SSBE, indicating that SSBE and LSBE have a similar oncogenic potential [55]. Between 1990 and 1999, the Japanese Society for Esophageal Diseases investigated 10,253 patients with esophageal cancer at 50 institutions across Japan and reported that BEA was present in 0.67%  (*n* = 69) of all patients, and 44.9% of the 69 adenocarcinoma cases originated from SSEE. This result again contradicts the clinical situation seen in Western countries and may be a characteristic of Barrett's adenocarcinoma unique to Japan. As described earlier, many SSBE cases in Japan are not accompanied by intestinal metaplasia, indicating that adenocarcinoma of the lower esophagus derives from two sources: intestinal metaplasia and gastric mucosa without intestinal metaplasia. Even though emphasis has been placed on intestinal metaplasia as the source of esophageal adenocarcinoma in Western countries, it is likely that adenocarcinoma originating from SSBE in Japan has a different background. With or without a clear difference in pathogenesis, epidemiological investigation of SSBE is not adequate in Japan because of the low number of cases, and therefore, more studies are needed to further assess the clinical situation.

## 8. Prevention of Barrett's Adenocarcinoma

A number of studies are currently being conducted on the prevention of BEA. GERD responds well to proton pump inhibitors (PPI), which suppress the secretion of stomach acid. However, these inhibitors do not significantly reduce the length of BE and have inconsistent cancer-preventive effects [56, 57]. Moreover, long-term administration of PPI increases the secretion of gastrin and facilitates oncogenesis via activation of cell proliferation and induction of cyclooxygenase 2 (COX2) [57]. A long-term follow-up study of BE cases is also underway, and the cancer-preventive effects of PPI are awaited with great interest.

COX2 is upregulated in inflamed tissues and promotes tumorigenesis via prostaglandin E_2_-mediated proliferation and angiogenesis. BE and BEA express COX2 at high levels, and a COX2 inhibitor has been shown to inhibit malignant conversion in an animal model. In addition, BEA is suppressed by COX2 selective inhibitors and nonsteroid anti-inflammatory drugs (NSAIDs), which inhibit both COX1 and COX2, making these agents powerful candidates for chemoprevention [56, 57]. However, because of their potential to damage tissue in the gastrointestinal tract, NSAIDs may cause peptic ulcers in the stomach and the duodenum in addition to the esophagus. For this reason, prospective cohort studies are needed to elucidate the efficacy of NSAIDs in the prevention of adenocarcinoma.

The recommendations for BE followup are as follows: once every 2-3 years if no dysplasia is observed in an endoscopic BE surveillance program, twice a year in low-grade dysplasia cases, and once every 3 months in high-grade dysplasia cases [58]. Random biopsy, in which tissue biopsies are taken at 2 cm intervals in four directions (if dysplasia is present, the interval should be 1 cm), has been regarded as the gold standard [59], and this is supported by a prospective study that demonstrated the superiority of random biopsy over other surveillance methods [60]. However, this method has been a subject of intense debate because of problems associated with time, cost, and safety.

Recently a large-scale cohort study was conducted in Denmark to investigate the incidence of adenocarcinoma and high-grade dysplasia in BE patients. During the median 5-year follow-up period, BE patients had a standardized incidence of 11.3% (8.8–14.4%) and an annual cancer incidence of 0.12%. The latter value was several times smaller than the baseline rate (0.5%) currently used in the guideline for endoscopic observation [61]. When taking the results of other studies on the cost effectiveness of the method and the quality of life of patients into consideration, we do not have a favorable view on endoscopic surveillance of BE patients with no dysplasia. Indeed, the results for the Denmark cohort have generally raised a question on the endoscopic surveillance of BE, despite the fact that the risk of BEA is 11.3 times higher in BE patients than in healthy individuals, and thus BE is clearly a powerful risk factor for BEA.

## 9. Conclusion

BE and BEA have been attracting attention in Japan because of the rapid increase in the incidence of BEA in Western countries. The prevalence of GERD—the cause of BE and BEA—is on the rise in Japan, as in Western countries, suggesting that both types of lesion will increase in the future. The clinical situation of BE is currently quite different from that in Western countries; however, this has not been investigated fully in Japan because of the low incidence of esophageal adenocarcinoma. To develop a surveillance program for BE unique to Japan, further studies are needed to elucidate the mechanisms of onset and the growth stages.

## References

[1] Cameron AJ (2002). Epidemiology of Barrett’s esophagus and adenocarcinoma. *Diseases of the Esophagus *.

[2] Hongo M, Shoji T (2003). Epidemiology of reflex disease and CLE in East Asia. *Journal of Gastroenterology*.

[3] Sampliner RE (1998). Practice guidelines on diagnosis, surveillance, and therapy of Barrett’s esophagus. *The American Journal of Gastroenterology*.

[4] Vieth M, Aida J, Takubo K (2009). Cardiac rather than intestinal-type background in endoscopic resection specimens of minute Barrett adenocarcinoma-reply. *Human Pathology*.

[5] Playford RJ (2006). New British Society of Gastroenterology (BSG) guidelines for the diagnosis and management of Barrett’s oesophagus. *Gut*.

[6] Riddell RH, Odze RD (2009). Definition of Barrett’s esophagus: time for a rethink—is intestinal metaplasia dead?. *The American Journal of Gastroenterology*.

[7] Armstrong D (2004). Review article: towards consistency in the endoscopic diagnosis of Barrett’s oesophagus and columnar metaplasia. *Alimentary Pharmacology & Therapeutics*.

[8] Sharma P, Dent J, Armstrong D (2006). The development and validation of an endoscopic grading system for Barrett’s esophagus: the Prague C & M criteria. *Gastroenterology*.

[9] Avidan B, Sonnenberg A, Schnell TG (2002). Hiatal hernia and acid reflux frequency predict presence and length of Barrett’s esophagus. *Digestive Diseases and Sciences*.

[10] Okita K, Amano Y, Takahashi Y (2008). Barrett’s esophagus in Japanese patients: its prevalence, form, and elongation. *Journal of Gastroenterology*.

[11] Fass R, Hell RW, Garewal HS (2001). Correlation of oesophageal acid exposure with Barrett’s oesophagus length. *Gut*.

[12] Ronkainen J, Aro P, Storskrubb T (2005). Prevalence of Barrett’s esophagus in the general population: an endoscopic study. *Gastroenterology*.

[13] Westhoff BC, Weston A, Cherian R (2004). Development of Barrett’s esophagus six months after total gastrectomy. *The American Journal of Gastroenterology*.

[14] Stoker DL, Williams JG (1991). Alkaline reflux oesophagitis. *Gut*.

[15] Kauer WKH, Peters JH, DeMeester TR (1995). Mixed reflux of gastric and duodenal juices is more harmful to the esophagus than gastric juice alone: the need for surgical therapy re- emphasized. *Annals of Surgery*.

[16] Caldwell MT, Lawlor P, Byrne PJ (1995). Ambulatory oesophageal bile reflux monitoring in Barrett’s esophagus. *British Journal of Surgery*.

[17] Wong NA, Wilding J, Bartlett S (2005). CDX1 is an important molecular mediator of Barrett’s metaplasia. *Proceedings of the National Academy of Sciences of the United States of America*.

[18] Kazumori H, Ishihara S, Rumi MAK, Kadowaki Y, Kinoshita Y (2006). Bile acids directly augment caudal related homeobox gene Cdx2 expression in oesophageal keratinocytes in Barrett’s epithelium. *Gut*.

[19] Jacobson BC, Somers SC, Fuchs CS (2006). Body mass index and symptoms of gastroesophageal reflex in woman. *The New England Journal of Medicine*.

[20] Herlihy KJ, Orlando RC, Bryson JC, Bozymski EM, Carney CN, Powell DW (1984). Barrett’s esophagus: clinical, endoscopic, histologic, manometric, and electrical potential difference characteristics. *Gastroenterology*.

[21] Cameron AJ, Lomboy CT (1992). Barrett’s esophagus: age, prevalence, and extent of columnar epithelium. *Gastroenterology*.

[22] Manabe N, Haruma K, Imamura H (2011). Does short-segment columnar-lined esophagus elongate during a mean follow-up period of 5. 7 years?. *Digestive Endoscopy*.

[23] Asayama M, Shibata M, Kondo Y (2005). Retrospective cohort study of chronological change of short-segment Barrett’s esophagus. *Digestive Endoscopy*.

[24] Sharma P, Morales TG, Bhattacharyya A (1997). Dysplasia in short-segment Barrett’s esophagus: a prospective 3-year follow-up. *The American Journal of Gastroenterology*.

[25] Spechler SJ (1992). Epidemiology and natural history of gastro-oesophageal reflex disease. *Digestion*.

[26] Sarr MG, Hamilton SR, Marrone GC (1985). Barrett’s esophagus: its prevalence and association with adenocarcinoma in patients with symptoms of gastroesophageal reflux. *The American Journal of Surgery*.

[27] Winters C, Spurling TJ, Chobanian SJ (1987). Barrett’s esophagus. A prevalent, occult complication of gastroesophageal reflux disease. *Gastroenterology*.

[28] Cameron AJ, Zinsmeister AR, Ballard DJ (1990). Prevalence of columnar-lined (Barrett’s esophagus). *Gastroenterology*.

[29] Cameron AJ, Lomboy CT (1992). Barrett’s esophagus: age, prevalence, and extent of columnar epithelium. *Gastroenterology*.

[30] Bonelli L (1993). Barrett’s esophagus: results of a multicentric survey. *Endoscopy*.

[31] Robinson M, Earnest D, Rodriguez-Stanley S (1998). Heartburn requiring frequent antacid use may indicate significant illness. *Archives of Internal Medicine*.

[32] Spechler SJ, Zeroogian JM, Antonioli DA, Wang HH, Goyal RK (1994). Prevalence of metaplasia at the gastro-oesophageal junction. *The Lancet*.

[33] Nandurkar S, Talley NJ (1999). Barrett’s esophagus: the long and the short of it. *The American Journal of Gastroenterology*.

[34] Johnston MH, Hammond AS, Laskin W, Jones DM (1996). The prevalence and clinical characteristics of short segments of specialized intestinal metaplasia in the distal esophagus on routine endoscopy. *The American Journal of Gastroenterology*.

[35] Hirota WK, Loughney TM, Lazas DJ, Maydonovitch CL, Rholl V, Wong RKH (1999). Specialized intestinal metaplasia, dysplasia, and cancer of the esophagus and esophagogastric junction: prevalence and clinical data. *Gastroenterology*.

[36] Amano Y, Kushiyama Y, Yuki T (2006). Prevalence of and risk factors for Barrett’s esophagus with intestinal predominant mucin phenotype. *Scandinavian Journal of Gastroenterology*.

[37] Kusano C, Gotoda T, Khor CJ (2008). Changing trends in the proportion of adenocarcinoma of the esophagogastric junction in a large tertiary referral center in Japan. *Journal of Gastroenterology and Hepatology*.

[38] Fujiwara Y, Arakawa T (2009). Epidemiology and clinical characteristics of GERD in the Japanese population. *Journal of Gastroenterology*.

[39] Raghunath A, Hungin APS, Wooff D, Childs S (2003). Prevalence of *Helicobacter pylori* in patients with gastro-oesophageal reflux disease: systematic review. *British Medical Journal*.

[40] Souza RF, Krishnan K, Spechler SJ (2008). Acid, bile, and CDX: the ABCs of making Barrett’s metaplasia. *American Journal of Physiology*.

[41] Koek GH, Sifrim D, Lerut T, Janssens J, Tack J (2008). Multivariate analysis of the association of acid and duodeno-gastro- oesophageal reflux exposure with the presence of oesophagitis, the severity of oesophagitis and Barrett’s oesophagus. *Gut*.

[42] Chen KH, Mukaisho KI, Sugihara H, Araki Y, Yamamoto G, Hattori T (2007). High animal-fat intake changes the bile-acid composition of bile juice and enhances the development of Barrett’s esophagus and esophageal adenocarcinoma in a rat duodenal-contents reflux model. *Cancer Science*.

[43] Fitzgerald RC (2006). Molecular basis of Barrett’s oesophagus and oesophageal adenocarcinoma. *Gut*.

[44] Everhart JE, Ruhl CE (2009). Burden of digestive diseases in the United States part I: overall and upper gastrointestinal diseases. *Gastroenterology*.

[45] El-Serag HB, Sonnenberg A (1998). Opposing time trends of peptic ulcer and reflux disease. *Gut*.

[46] Rokkas T, Pistiolas D, Sechopoulos P (2007). Relationship between *Helicobactor pylori* infection and esophageal neoplasia: a meta-analysis. *Clinical Gastroenterology and Hepatology*.

[47] McArdle JE, Lewin KL, Randell G (1992). Distribution of dysplasias and early invasive carcinoma in Barrett’s esophagus. *Human Pathology*.

[48] Shaheen NJ, Crosby MA, Bozymski EM, Sandler RS (2000). Is there publication bias in the reporting of cancer risk in Barrett’s esophagus?. *Gastroenterology*.

[49] Sikkema M, de Jonge PJ, Steyerberg EW (2010). Risk of esophageal adenocarcinoma and mortality in patients with Barrett’s esophagus: a systematic review and metaanalysis. *Clinical Gastroenterology and Hepatology*.

[50] Spechler SJ, Sharma P, Souza RF (2011). American Gastroenterological Association medical position statement on the management of Barrett’s esophagus. *Gastroenterology*.

[51] Montgomery E, Goldblum JR, Greeson JK (2001). Dysplasia as s predictive marker for invasive carcinoma in Barrett’s esophagus: a follow-up study based on 138 cases from a diagnostic variability study. *Human Pathology*.

[52] Weston AP, Sharma P, Mathur S (2004). Risk stratification of Barrett’s esophagus: updated prospective multivariate analysis. *The American Journal of Gastroenterology*.

[53] Srinivas G, Patric EY, Benjamin RA Relationship between Barrett’s esophagus (BE) length and risk of high grade dysplasia (HGD) and esophageal adenocarcinoma (EAC) in patient with non dysplastic Barrett’s esophagus results from a large multicenter cohort.

[54] Wani S, Falk G, Hall M (2011). Patients with nondysplastic Barrett’s esophagus have low risks for developing dysplasia or esophageal adenocarcinoma. *Clinical Gastroenterology and Hepatology*.

[55] Schnell TG, Sontag SJ, Chejfec G (1992). Adenocarcinoma arising in tongues or short segments of Barrett’s esophagus. *Digestive Diseases and Sciences*.

[56] Badreddine RJ, Wang KK (2010). Barrett esophagus: an update. *Nature Reviews Gastroenterology & Hepatology*.

[57] Prasad GA, Bansal A, Sharma P (2010). Predictors of progression in Barrett’s esophagus: current knowledge and future directions. *The American Journal of Gastroenterology*.

[58] Sampliner RE (1998). Practice guidelines on the diagnosis, surveillance, and therapy of Barrett’s esophagus. *The American Journal of Gastroenterology*.

[59] Sampliner RE (2002). ‘The Practice Parameters Committee of the American College of Gastroenterology’, updated guidelines for the diagnosis, surveillance, and therapy of Barrett’s esophagus. *The American Journal of Gastroenterology*.

[60] Egger K, Werner M, Meining A (2003). Biopsy surveillance is still necessary in patients with Barrett’s esophagus despite new endoscopic imaging techniques. *Gut*.

[61] Hvid-Jensen F, Pedersen L, Drewes AM (2011). Incidence of Adenocarcinoma among patients with Barrett's Esophagus. *The New England Journal of Medicine*.

